# Origin and Spread of *Bos taurus*: New Clues from Mitochondrial Genomes Belonging to Haplogroup T1

**DOI:** 10.1371/journal.pone.0038601

**Published:** 2012-06-07

**Authors:** Silvia Bonfiglio, Catarina Ginja, Anna De Gaetano, Alessandro Achilli, Anna Olivieri, Licia Colli, Kassahun Tesfaye, Saif Hassan Agha, Luis T. Gama, Federica Cattonaro, M. Cecilia T Penedo, Paolo Ajmone-Marsan, Antonio Torroni, Luca Ferretti

**Affiliations:** 1 Dipartimento di Biologia e Biotecnologie “L. Spallanzani", Università di Pavia, Pavia, Italy; 2 Molecular Biology Group, Instituto Nacional de Recursos Biológicos, INIA, and Faculty of Sciences, Environmental Biology Centre, University of Lisbon, Lisbon, Portugal; 3 Dipartimento di Biologia Cellulare e Ambientale, Università di Perugia, Perugia, Italy; 4 Centro di Ricerca sulla Biodiversità e sul DNA Antico – BioDNA and Istituto di Zootecnica, Università Cattolica del Sacro Cuore, Piacenza, Italy; 5 Microbial, Cellular and Molecular Biology Program Unit, Faculty of Life Sciences, University of Addis Ababa, Addis Ababa, Ethiopia; 6 Department of Animal Production, Faculty of Agriculture, Ain Shams University, Cairo, Egypt; 7 Faculdade de Medicina Veterinária, Universidade Técnica de Lisboa, Lisbon, Portugal; 8 Applied Genomics Institute (IGA), Udine, Italy; 9 Veterinary Genetics Laboratory, University of California Davis, Davis, California, United States of America; University of Florence, Italy

## Abstract

**Background:**

Most genetic studies on modern cattle have established a common origin for all taurine breeds in the Near East, during the Neolithic transition about 10 thousand years (ka) ago. Yet, the possibility of independent and/or secondary domestication events is still debated and is fostered by the finding of rare mitochondrial DNA (mtDNA) haplogroups like P, Q and R. Haplogroup T1, because of its geographic distribution, has been the subject of several investigations pointing to a possible independent domestication event in Africa and suggesting a genetic contribution of African cattle to the formation of Iberian and Creole cattle. Whole mitochondrial genome sequence analysis, with its proven effectiveness in improving the resolution of phylogeographic studies, is the most appropriate tool to investigate the origin and structure of haplogroup T1.

**Methodology:**

A survey of >2200 bovine mtDNA control regions representing 28 breeds (15 European, 10 African, 3 American) identified 281 subjects belonging to haplogroup T1. Fifty-four were selected for whole mtDNA genome sequencing, and combined with ten T1 complete sequences from previous studies into the most detailed T1 phylogenetic tree available to date.

**Conclusions:**

Phylogenetic analysis of the 64 T1 mitochondrial complete genomes revealed six distinct sub-haplogroups (T1a–T1f). Our data support the overall scenario of a Near Eastern origin of the T1 sub-haplogroups from as much as eight founding T1 haplotypes. However, the possibility that one sub-haplogroup (T1d) arose in North Africa, in domesticated stocks, shortly after their arrival from the Near East, can not be ruled out. Finally, the previously identified “African-derived American" (AA) haplotype turned out to be a sub-clade of T1c (T1c1a1). This haplotype was found here for the first time in Africa (Egypt), indicating that it probably originated in North Africa, reached the Iberian Peninsula and sailed to America, with the first European settlers.

## Introduction

The domestication of the wild aurochs (*Bos primigenius*) ∼10 thousand years (ka) ago was a major element of the Neolithic transition and a fundamental step in human development, contributing to the rise of larger settlements and more stratified societies [1], [2]. From a genetic point of view, animal domestication can be reconstructed through phylogeographic analyses of both nuclear and mitochondrial genomic data [3]. Early molecular and evolutionary studies on cattle have focused on mitochondrial DNA (mtDNA), in particular on short segments of its control region [4], [5], [6]. However, mtDNA control-region variation is often characterized by high levels of recurrent mutations and reversions, thus blurring the structure of the phylogenetic tree and making the distinction between some important branches within the tree virtually impossible [7].

As for *Bos taurus* cattle, recent studies carried out at the whole mitochondrial genome level have overcome this limitation, showing that macro-haplogroup T is made up of two sister clades, T1′2′3 and T5 [8], [9], with the former encompassing the initially defined haplogroups T1, T2 and T3 [5], and T4 [6], clustering within T3 [8]. All T haplogroups most likely originated and underwent domestication in the Fertile Crescent from where they spread with the diffusion of *B. taurus* domestic herds [5], [8], [10]. However, haplogroups T1–T5 do not represent the totality of mtDNAs from modern taurine breeds. Analyses of entire mtDNA sequences have shown that a small subset belongs to three other rare haplogroups (P, Q and R). Haplogroup Q is most likely of Near Eastern origin, but P and R mtDNAs probably derive from European populations of wild aurochsen [8], [9]. Interestingly, some recent studies have also raised the possibility that local and secondary events of *B. primigenius* domestication might have occurred in Italy [11], [12].

Studies carried out on human mtDNA have shown that, after having defined the major branches of the phylogeny and the deep relationships between major haplogroups, the complete sequencing approach is also an extremely powerful tool to dissect haplogroups into sub-haplogroups of younger age, whose spatial frequency patterns might in turn be correlated with prehistoric and historical migratory events [7].

The bovine T1 haplogroup is of particular interest, as it possesses peculiar phylogeographic features. Although present in Middle Eastern and Anatolian breeds, it is quite common in breeds from southern Europe (Portugal, Spain, Greece and Italy), and is almost fixed in African cattle [4], [11], [13], [14], [15]. Due to this distribution, haplogroup T1 has been the subject of numerous papers investigating both the possibility of an independent domestication event in Africa [4], [14], and the genetic influence of African cattle in the formation of Iberian and Creole breeds [13], [16], [17], [18], [19], [20], [21], [22].

The scenario of a domestication event in Africa has been dismissed mainly on the basis of mitochondrial genome sequencing data [8] showing that the nodal T1 genome was only one mutation away (np 16113) from the T1′2′3 node and only two mutations away (nps 16113 and 16255) from the T3 node in the phylogeny. Thus, domestication of T1 in Africa would have required that the *B. primigenius* populations of North Africa, during their allopatric evolution in Africa, had accumulated no sequence variation in their entire mtDNA (except the T1 marker 16113) relative to the Near Eastern stocks – an unlikely event.

As for the role of African cattle in the formation of certain European and American breeds, a distinctive control-region mutational motif (16050–16053–16113–16122–16139–16196–16255) has allowed the identification of a T1 sub-haplogroup, initially termed “African-derived American" (AA) in South American cattle breeds [16], in subjects of Spanish Retinta [16], [17] and Lidia breeds [21], but not in African breeds, thus raising the possibility that Iberian, rather than North African cattle, were the main genetic source of the American taurine breeds.

In this study, to obtain more information concerning the phylogeny of haplogroup T1, its origin and the processes that led to its current geographical and breed distribution, we analyzed a total of 64 T1 mtDNA genomes (54 reported here for the first time; GenBank records JN817298-JN817351) from a wide variety of cattle breeds from Europe, Africa and the Americas. Our analysis allowed the detection of six major T1 sub-branches, confirmed the overall Near-Eastern origin of haplogroup T1, but also raised the possibility of a local African origin for one of its sub-clades.

## Results

### The phylogeny of haplogroup T1

**Figure 1:**
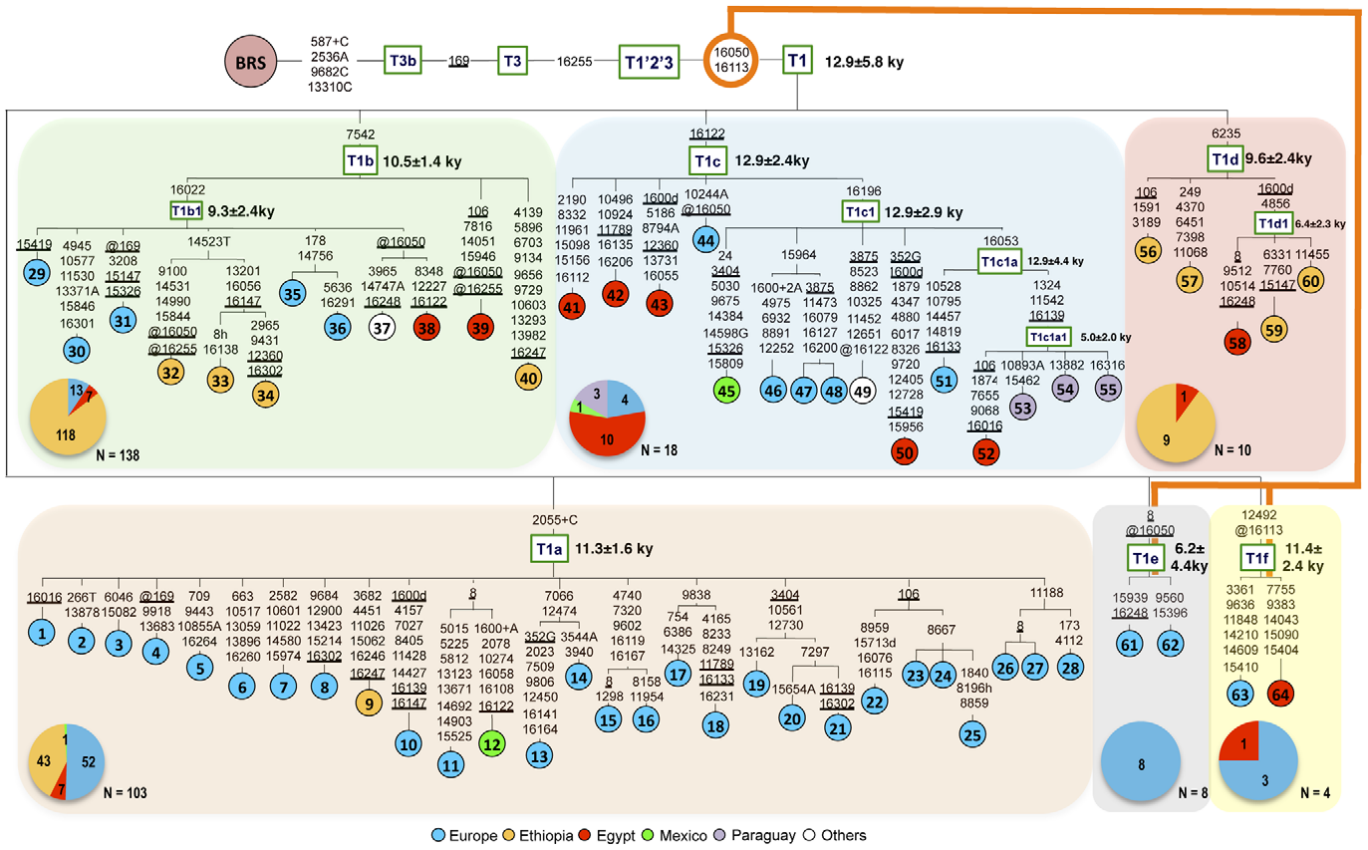
Tree of Complete Bovine mtDNA Sequences Belonging to Haplogroup T1. This tree was built as previously described [8], [11]. The position of the Bovine Reference Sequence (BRS) [23] is indicated for reading off-sequence motifs. Divergence time estimates are those obtained using ML as reported in Table 3. Branches display mutations with numbers according to the BRS; they are transitions unless a base is explicitly indicated for transversions (to A, G, C, or T) or a suffix for indels (+, d) and should be read as if the BRS was an artificial root. Recurrent mutations are underlined; back mutations at positions that separate the T1 tree from the BRS are prefixed with the superscript @. Note that the reconstruction of recurrent mutations in the control region is ambiguous in a number of cases. Heteroplasmy is marked with a suffix (h). The numbering of sequences is the same as in Table 2. The orange line connecting T1e and T1f to nps 16050 and 16113 reflects the uncertainty of their classification as quasi-sister taxa of sub-haplogroups T1a to T1d and implies the possibility that either one or the other or both might well descend from a T1′2′3′ – T1 intermediate (see Discussion for more details). Note that a potential affiliation of sequence #44 within sub-haplogroup T1e should be also considered (see Results). The pie charts summarize the typing results of the survey performed on our entire dataset of 281 T1 mtDNAs (Table 1) with diagnostic T1 sub-haplogroups markers. The numbers of mtDNAs for each sub-haplogroup are shown and include the 54 mtDNAs completely sequenced in this study, but not those previously published (#3, 4, 10, 22, 23, 29, 37, 46, 49 and 62) (see also Table 2). Colors in the pie charts indicate geographical origins.

To identify mtDNAs belonging to haplogroup T1, we took advantage of its diagnostic mutational motif in the control region (16050–16113–16255) relative to the bovine reference sequence (BRS) [23]. Thus we sequenced the control region of more than two thousand mtDNA samples from European, African and American cattle breeds. This allowed the identification of 281 T1 mtDNAs (Table 1; Table S1). Fifty-four, selected on the basis of control-region data and geographical origin in order to include the widest possible range of internal haplogroup variation, were completely sequenced and, together with ten T1 mitochondrial genomes recovered from the literature (Table 2), were employed to build a detailed phylogeny of haplogroup T1 (Figure 1). Among the 64 complete sequences, 55 harboured both mutations at nps 16050 and 16113, relative to T1′2′3 node, but nine (#32, 37–39, 44, 61–64) lacked either one or the other, most probably due to independent reversion events.

**Table 1:** List of the 281 T1 mtDNAs included in our dataset. Geographical origins, breeds and sub-haplogroup affiliations are indicated.

Country	Breed	Frequencies of T1 sub-haplogroups (and their diagnostic marker mutations)						Total
		T1a	T1b	T1c	T1d	T1e	T1f	
		2055+C	7542	16122	6235	8 @16050	12492 @16113	
Europe								
Italy	Agerolese	4	0	0	0	0	0	4
Italy	Calvana	0	0	0	0	2	0	2
Italy	Chianina	9	4	0	0	6	0	19
Italy	Cinisara	10	2	1	0	0	0	13
Italy	Italian Brown	1	0	0	0	0	0	1
Italy	Italian Podolian	5	0	0	0	0	3	8
Italy	Italian Red Pied	0	1	0	0	0	0	1
Italy	Marchigiana	6	5	0	0	0	0	11
Italy	Maremmana	3	0	0	0	0	0	3
Italy	Modicana	1	0	0	0	0	0	1
Italy	Reggiana	2	0	0	0	0	0	2
Italy	Romagnola	8	0	1	0	0	0	9
France	Limousin	3	0	0	0	0	0	3
Portugal	Alentejana	0	0	2	0	0	0	2
Turkey	Grey Steppe	0	1	0	0	0	0	1
*Europe total*		*52 (65.0%)*	*13 (16.3%)*	*4 (5.0%)*	*0 (0%)*	*8 (10.0%)*	*3 (3.7%)*	*80 (100%)*
Africa								
Egypt	Domiaty	2	2	3	1	0	0	8
Egypt	Khaleit	0	2	4	0	0	0	6
Egypt	Menofi	5	3	3	0	0	1	12
*Egypt total*		*7 (26.9%)*	*7 (26.9%)*	*10 (38.5%)*	*1 (3.8%)*	*0 (0%)*	*1 (3.8%)*	*26 (100%)*
Ethiopia	Abigar	6	4	0	1	0	0	11
Ethiopia	Arsi	5	25	0	2	0	0	32
Ethiopia	Boran	8	22	0	5	0	0	35
Ethiopia	Guraghe	1	10	0	0	0	0	11
Ethiopia	Bark	3	8	0	0	0	0	11
Ethiopia	Horro	14	39	0	1	0	0	54
Ethiopia	Sheko	6	10	0	0	0	0	16
*Ethiopia total*		*43 (25.3%)*	*118 (69.4%)*	*0 (0%)*	*9 (5.3%)*	*0 (0%)*	*0 (0%)*	*170 (100%)*
*Africa total*		*50 (25.5%)*	*125 (63.8%)*	*10 (5.1%)*	*10 (5.1%)*	*0 (0%)*	*1 (0.5%)*	*196 (100%)*
America								
Mexico	Baja California Creole	0	0	1	0	0	0	1
Mexico	Chihuahua Creole	1	0	0	0	0	0	1
Paraguay	Pampa Chaqueño Creole	0	0	3	0	0	0	3
*America total*		*1 (20.0%)*	*0 (0%)*	*4 (80.0%)*	*0 (0%)*	*0 (0%)*	*0 (0%)*	*5 (100%)*
***TOTAL***		***103 (36.7%)***	***138 (49.1%)***	***18 (6.4%)***	***10 (3.6%)***	***8 (2.8%)***	***4 (1.4%)***	***281 (100%)***

**Table 2:** List of T1 complete mitochondrial genomes analyzed in this study.

ID# a	Sample ID	Sub-haplogroup	Breed	GenBank ID	Reference
1	PER10 b	T1a	Agerolese	JN817341	This study
2	CHI336 b	T1a	Chianina	JN817313	This study
3	Bos9	T1a	Maremmana	EU177844	[8]
4	Bos4	T1a	Italian Podolian	EU177843	[8]
5	MCG469 b	T1a	Marchigiana	JN817339	This study
6	CH34 c	T1a	Italian Brown	JN817312	This study
7	CHI575 b	T1a	Chianina	JN817316	This study
8	ROM558 b	T1a	Romagnola	JN817347	This study
9	AR22 c	T1a	Arsi	JN817303	This study
10	CB86	T1a	Angus mix	GU947020	[28]
11	MCG378 b	T1a	Marchigiana	JN817336	This study
12	CCH01 c	T1a	Chihuahua Creole	JN817308	This study
13	MCG452 b	T1a	Marchigiana	JN817338	This study
14	CIN15 b	T1a	Cinisara	JN817318	This study
15	CIN11 d	T1a	Cinisara	JN817317	This study
16	PER5 b	T1a	Agerolese	JN817340	This study
17	CHI397 b	T1a	Chianina	JN817314	This study
18	LMI50 b	T1a	Limousin	JN817331	This study
19	MAR12 c	T1a	Maremmana	JN817333	This study
20	PER17 b	T1a	Agerolese	JN817342	This study
21	MCG383 b	T1a	Marchigiana	JN817337	This study
22	Bos17	T1a	Chianina	EU177846	[8]
23	Bos7	T1a	Chianina	EU177845	[8]
24	POD41 b	T1a	Italian Podolian	JN817344	This study
25	ROM242 b	T1a	Romagnola	JN817345	This study
26	MAR10 c	T1a	Maremmana	JN817332	This study
27	CHI481 b	T1a	Chianina	JN817315	This study
28	MCG363 b	T1a	Marchigiana	JN817335	This study
29	Bos2	T1b1	Cinisara	EU177842	[8]
30	MCG358 b	T1b1	Marchigiana	JN817334	This study
31	CINL14 d	T1b1	Cinisara	JN817320	This study
32	AR18 c	T1b1	Arsi	JN817302	This study
33	S13 b	T1b1	Sheko	JN817349	This study
34	B1 b	T1b1	Boran	JN817305	This study
35	CHI425 b	T1b1	Chianina	JN817350	This study
36	MCG489 b	T1b1	Marchigiana	JN817351	This study
37	FC13	T1b1	Beef cattle	DQ124399	Unpublished
38	EG19 c	T1b1	Domiaty	JN817324	This study
39	EG27 c	T1b	Menofi	JN817327	This study
40	S8 c	T1b	Sheko	JN817348	This study
41	EG23 b	T1c	Menofi	JN817326	This study
42	EG22 c	T1c	Menofi	JN817325	This study
43	EG28 c	T1c	Menofi	JN817328	This study
44	CINL5B b	T1c^e^	Cinisara	JN817319	This study
45	CBC16 c	T1c1	Baja California Creole	JN817307	This study
46	Bos24	T1c1	Friesian	EU177847	[8]
47	ALT9301 c	T1c1	Alentejana	JN817301	This study
48	ALT9280 c	T1c1	Alentejana	JN817300	This study
49	Bos37	T1c1	Iraqi	EU177848	[8]
50	EG18 b	T1c1	Domiaty	JN817323	This study
51	ROM480 b	T1c1a	Romagnola	JN817346	This study
52	EG12 c	T1c1a1	Domiaty	JN817322	This study
53	CCQ40 c	T1c1a1	Pampa Chaqueño Creole	JN817311	This study
54	CCQ3 c	T1c1a1	Pampa Chaqueño Creole	JN817309	This study
55	CCQ31 c	T1c1a1	Pampa Chaqueño Creole	JN817310	This study
56	A8 c	T1d	Abigar	JN817298	This study
57	ADT23 c	T1d	Boran	JN817299	This study
58	EG11 b	T1d1	Domiaty	JN817321	This study
59	AR29 c	T1d1	Arsi	JN817304	This study
60	H23 c	T1d1	Horro	JN817330	This study
61	CAL24 c	T1e	Calvana	JN817306	This study
62	Bos6	T1e	Chianina	EU177841	[8]
63	POD16N c	T1f	Italian Podolian	JN817343	This study
64	EG36 b	T1f	Menofi	JN817329	This study

a ID numbers are those reported in the phylogeny of Figure 1.

b Illumina sequencing.

c Sanger sequencing.

d Completely sequenced with both Illumina and Sanger approaches.

e A potential affiliation within sub-haplogroup T1e should also be considered.

### The dissection of haplogroup T1 into sub-haplogroups

The T1 mitochondrial genomes of Figure 1 clustered within six sub-haplogroups: T1a, T1b, T1c, T1d, T1e and T1f. The first four sub-haplogroups comprise 60 mtDNAs and are each defined by a diagnostic marker mutation: the insertion 2055+C for T1a, the transition at np 7542 for T1b, the transition at np 16122 for T1c and the transition at np 6235 for T1d. Two sequences (#61, 62) belonged to T1e, defined by a transition at np 8 and a reversion at np 16050. Finally, two sequences (#63, 64) formed T1f, which is characterized by a transition at np 12492 and a reversion at np 16113.

Sub-haplogroup T1a is the most represented in our phylogeny with 26 distinct haplotypes (28 mtDNAs) and a pronounced star-like structure. Most of the T1a sequences in Figure 1 are from European breeds, but one mtDNA from Ethiopia (Arsi) and one from Mexico (Chihuahua Creole) are also present (Figure 1; Table 2).

Sub-haplogroup T1b comprises twelve sequences, of which ten are new and two were previously reported. This sub-haplogroup harbours both European (five from Italy) and non-European (four Ethiopians, two ians and one from Korea) samples (Figure 1; Table 2). The majority of the T1b sequences (ten out of 12) clusters into a sub-clade, termed T1b1, which is defined by the control-region transition at np 16022.

The third sub-haplogroup, T1c, is defined by the control-region mutation at np 16122 and comprises 15 sequences from a wide range of geographical areas, including Near East, Africa, Europe and the Americas. T1c is the most structured sub-haplogroup, with a major sub-branch – T1c1 – that includes eleven sequences and two further sub-branches: T1c1a and T1c1a1. T1c1 membership is marked by the transition at np 16196, while the sub-clade T1c1a is characterized by the additional transition at np 16053. Four T1c1a mtDNAs (one Domiaty from Egypt and three Pampa Chaqueño Creoles from Paraguay) harboured a distinctive combination of transitions at nps 1324, 11542 and 16139 forming the sub-clade T1c1a1. This sub-clade was previously described in the literature as “haplogroup AA" [16], [17] on the basis of its control-region motif (16050–16113–16122–16139–16196–16255).

Note that sequence #44 was classified within sub-haplogroup T1c because of the transition at np 16122 (Figure 1). However, this control-region position is prone to parallel mutational events (see sequences #12 and #38). Therefore, when taking into account that sequence #44 harbours the reversion at np 16050, a potential affiliation within sub-haplogroup T1e should also be considered.

Five mitochondrial genomes are members of the sub-haplogroup T1d. They are all of African origin: four from Ethiopia and one from Egypt. Three mtDNAs (#58–60) clustered within sub-clade T1d1, that is characterized by the transition at np 4856 and the deletion of the consensus nucleotide at np 1600 (Figure 1; Table 2).

The remaining four T1 sequences (#61–64) did not fit into any of the four sub-haplogroups described above. Indeed they lacked the diagnostic mutations of T1a (2055+C), T1b (7542), T1c (16122) and T1d (6235) and formed two separate branches (T1e and T1f), each defined by a specific combination of two mutations. Sub-haplogroup T1e is defined by the control-region transition at np 8 and the lack of the diagnostic mutation at np 16050, while sub-haplogroup T1f is characterized by the transition at np 12492 and lacks 16113, the other diagnostic T1 mutation.

### Age estimates of T1 and its sub-haplogroups

The maximum-likelihood (ML) divergence for the entire T1 haplogroup based on the 64 complete mtDNA sequences is 0.00026±0.00005 substitutions per site. This corresponds to a divergence time of 12.5±2.3 ka according to the mutation rate proposed [8] (Table 3). The ML divergences for sub-haplogroups T1a, T1b, T1c, T1d and T1f are not much lower and very close to each other, with values of substitutions per site ranging from 0.00026±0.00007 (T1c) to 0.00019±0.00005 (T1d), corresponding to divergence times between 12.5±3.6 ka and 9.4±2.3 ka, respectively (Figure 1). These divergence times are confirmed when the average distance of the haplotypes from the roots of T1 and its major sub-haplogroups (ρ-statistics) are computed (Table 3). In this case, the time to the most recent common ancestor for T1 as a whole is 11.6±1.1 ka. As for T1a, T1b, T1c and T1d, ρ age estimates are between 12.7±2.0 ka for T1c and 9.5±2.9 ka for T1d. The ρ value observed for T1f (17.4±5.3 ka) probably represents an overestimate due to the low sample size (only two sequences). A similar consideration applies for the ρ age estimate of T1e (3.2±2.2 ka), which for the same reason is instead probably underestimated.

**Table 3:** Divergence values and time estimates of mtDNA haplogroup T1 and its subclades obtained by using maximum likelihood (ML) and ρ statistics.

Haplogroups/ Sub-haplogroups	No. of mtDNAs^a^	Maximum Likelihood				ρ^b^ Statistics			
		Substitutions per site	S.E.	T (ka)^c^	± ΔT (ka)	ρ	σ	T (ka)^c^	± ΔT (ka)
**T1**	64	0.00026	0.00005	12.5	2.3	3.656	0.340	11.6	1.1
**>T1a**	28	0.00023	0.00003	11.2	1.6	3.214	0.413	10.2	1.3
**>T1b**	12	0.00021	0.00005	10.2	2.3	3.083	0.571	9.8	1.8
**>>T1b1**	10	0.00019	0.00005	9.3	2.4	2.600	0.600	8.2	1.9
**>T1c**	15	0.00026	0.00007	12.5	3.6	4.000	0.625	12.7	2.0
**>>T1c1**	11	0.00026	0.00006	12.5	2.8	4.272	0.787	13.6	2.5
**>>>T1c1a**	5	0.00026	0.00013	12.5	6.3	3.600	1.296	11.4	4.1
**>>>>T1c1a1**	4	0.00010	0.00004	4.9	2.0	1.500	0.375	4.8	1.2
**>T1d**	5	0.00019	0.00005	9.4	2.3	3.000	0.917	9.5	2.9
**>>T1d1**	3	0.00013	0.00005	6.3	2.2	2.000	0.816	6.3	2.6
**>T1e**	2	0.00013	0.00009	6.3	4.5	1.000	0.707	3.2	2.2
**>T1f**	2	0.00023	0.00005	11.0	2.4	5.500	1.658	17.4	5.3

a These correspond to the T1 complete mtDNA sequences shown in Figure 1. Additional information regarding each mtDNA is provided in Table 2.

b Average number of base substitutions in the mtDNA coding region (between nps 364 and 15791) from the ancestral sequence type.

c Estimate of the time to the most recent common ancestor of each clade, using a mutation rate estimate of 3,172 years per substitution in the whole coding region (15,428 bp) [8].

It is worth to note that T1c1 and T1c1a, two clades within T1c, are both extremely divergent with substitutions per site and ρ values virtually identical to those estimated for the entire T1c and the other major sub-haplogroups (Figure 1). A similar situation occurs for T1b1 whose divergence overlaps that of T1b (Table 3). These findings indicate that the founding haplotypes of T1a, T1b, T1c, T1d and T1f might all have expanded roughly at the same time, and that the founding haplotypes of T1b1, T1c1 and T1c1a were also probably involved in the same expansion event. If this process corresponds to the domestication event, it would imply that at least eight different T1 haplotypes might have undergone domestication.

### Sub-haplogroup classification of our entire dataset of T1 samples and geographic distribution

The incidence of sub-haplogroups T1a-T1f among our 281 T1 samples was investigated by screening for the diagnostic markers of each sub-haplogroup (Table 1). The analysis revealed that 52 (65%) European T1 samples (eleven breeds) and 50 (25.5%) African samples (nine breeds) belonged to T1a, with frequencies in Ethiopia and Egypt that were virtually identical (25.3% vs 26.9%) (Figure 1, “pie charts").

Sub-haplogroup T1b encompassed 13 (16.3%, five breeds) and 125 (63.8%, ten breeds) European and African T1 samples, respectively, showing a much higher frequency in Ethiopia (69.4%) than in Egypt (26.9%), thus raising the possibility of serial founder events in the formation of Ethiopian breeds. Sub-haplogroup T1c was observed in only four (5%) European subjects (one Cinisara, two Alentejana and one Romagnola) and their mtDNAs underwent complete sequencing. This sub-haplogroup is very common in Egypt (10 subjects; 38.5%), but completely absent in the Ethiopian breeds. Sub-haplogroup T1d appears instead to be completely absent in Europe and only present in Africa (3.8% in Egypt and 5.3% in Ethiopia). This finding is the exact opposite of that observed for T1e, which is absent in Africa but not uncommon in Italian breeds (10.0%), in particular in the Calvana and Chianina breeds. Finally, sub-haplogroup T1f was only reported in four subjects (three from Europe and one from Egypt) with an overall frequency within T1 of 1.4%.

As for the only five American samples included in the dataset, they were all completely sequenced. One turned out to be a member of T1a while the remaining four clustered into T1c (Figure 1).

To better evaluate the geographical distribution of T1 and some of its sub-haplogroups, a GenBank survey of bovine mtDNA control regions was also performed (Table S2 and Data S1). The classification into haplogroup T1 was based on the presence of the diagnostic motif 16050–16113–16255. Overall, 752 T1 mtDNAs were retrieved from GenBank. As observed in the T1 samples that we analyzed, not all GenBank sequences carried the complete control-region mutational motif of T1 (649 out of 752). Haplogroup T1 was found in breeds from several countries and geographical areas. Most of the retrieved mtDNAs were from Africa (more than 50%) and Latin America (∼25%), but also Europe was represented (∼16%), especially South and South-western countries, and a few were from the Near East (Israel, Iraq) and Eastern Asia (China, Japan, Korea).

Almost all T1 sub-haplogroups are defined by diagnostic mutations located in the mtDNA coding region. Thus, control-region sequences are generally not informative for sub-haplogroup affiliation. Possible exceptions are T1b1, with its control-region mutation at np 16022, and the derivatives of T1c, with control-region mutational motifs 16122-16196 (T1c1), 16053–16122–16196 (T1c1a) and 16053–16122–16139–16196 (T1c1a1).

A survey for the mutation at np 16022 in the GenBank control-region entries (Table S2), reveals that only 334 out 752 sequences span np 16022. Among those, 131 carry the 16022 transition and can hence be considered putative T1b1, although the absence of coding-region information does not allow to rule out misclassifications. The majority (87) of these putative T1b1 are observed in African samples (82 from Ethiopia and 5 from Kenya). The others are found in southern European breeds (Portugal, Spain), American Creole cattle (Colombia, Mexico) and one in a local Chinese breed, Zhaotong.

Regarding sub-haplogroups T1c1, T1c1a and T1c1a1, GenBank data suggest that the nodal motif of T1c1 (16122–16196) is rather uncommon. In addition to the few mtDNAs from Iraq, Egypt, Italy, Portugal and Mexico reported in Figure 1, it was detected also in local breeds from Morocco, Tunisia and Libya. Its derivative motif 16053–16122–16196 (T1c1a), illustrated by sequence #51 (Romagnola, Italy) in Figure 1, was found only in two Cuban Creole and two Tunisian Blonde mtDNAs. T1c1a1, which was previously termed “AA", is the most recent sub-branch of T1c, and appears instead to be rather frequent (60 mtDNAs in Table S2). MtDNAs with the distinguishing motif 16053–16122–16139–16196 are indeed commonly reported in Mexico, Guadeloupe, St. Lucia, Brazil, Argentina and Paraguay, most likely due to a major founder event associated with the post-Columbian diffusion of cattle in Central and South America. The Egyptian sample #52 of Figure 1 is one of the very few Old World T1c1a1 mtDNAs detected until now. Other rare occurrences were previously reported in the Retinta and Lidia breeds of Spain [16], [17], [21].

## Discussion

### The updated T1 phylogenetic tree

The T1 phylogenetic tree described in this paper is based on a much wider number of complete mitochondrial genome sequences, 64 in total (Figure 1), compared to previous reports [8]. The haplogroup is now quite structured and can be split into six sub-haplogroups, named T1a to T1f. Moreover, the transitions at nps 16113 and 16050, previously used to define the single sub-haplogroup T1a [8], are now moved to the basal motif of T1, as originally described [5]. Sequences #32, 37–39, 44, 61–62, and 63–64 (Figure 1) represent exceptions to this T1 definition. Despite the reversion at np 16050, sequences #32 and #37–39 cluster within T1b, since they possess the diagnostic transition at np 7542, while sequence #44 is included into T1c due to the transition at np 16122. On the contrary, sequences # 61–62 and # 63–64 carry no diagnostic mutations of haplogroups T1a–T1d and clustered into separate sub-clades (T1e and T1f). However, we are aware of the ambigous grouping of one subject (#44), suggested as belonging to T1c in Figure 1. Infact due to the unstable mutation at np 16122, and the reversion at np 16050, that subject could possibly be part of sub-haplogroup T1e.

It should be noted that the lack of the 16050 mutation in T1e and that of the 16113 mutation in T1f raise the possibility of an alternative topology for these haplogroups (Figure 1). They could have both branched independently between the T1′2′3′ and T1 nodes prior to the occurrence of the 16050 mutation in the case of T1e and of the 16113 mutation in the case of T1f. The latter seems the most likely candidate for a pre-T1 split-off, mainly because nucleotide position 16113, in contrast to np 16050, does not appear to be much prone to reversions (Figure 1). We may never know the true topology of haplogroup T1. However, since this does not really affect a reconstruction of *Bos taurus* history mainly based on the phylogeography of the sub-haplogroups of T1, we suggest the tree in Figure 1, with T1e and T1f as quasi-sister taxa of T1a to T1d, as an operational view of the T1 phylogeny.

### The origin of haplogroup T1

The analysis of 64 entire mtDNA sequences still support the view that T1 underwent domestication in the Near East, like the other T haplogroups. Indeed, despite the identification of numerous novel polymorphisms that revealed the existence of six T1 sub-haplogroups (Figure 1), T1 still remains only two control-region mutations away (16050, 16113) from the node T1′2′3′. The observed geographic distributions of the six T1 sub-haplogroups and their coalescence time, which is generally comprised within the range of ∼10–13 ka, the upper limit for the domestication of *B. primigenius*, also lend support to an original domestication in the Near East and a later spread along the Neolithic migration routes of human populations. Sub-haplogroup T1e shows a more recent coalescence time (6.2±4.4 ka), but its estimate may be imprecise, due to the small number of individuals falling in this sub-clade (only two) and the few mutations revealed. Moreover, sub-haplogroups T1a, T1b and T1c, which represent the vast majority of the T1 clade, are clearly widespread in Europe, Africa and Asia, as shown by the analysis of complete sequences and by additional evidence from the entries retrieved from GenBank or belonging to our T1 dataset.

Overall our data indicate that 7–8 possible founding T1 sequences, i.e. at least 7–8 unrelated auroch females, might have undergone domestication at approximately the same time at the original domestication site(s). These sequences include most of the nodal haplotypes of the six sub-haplogroups plus the haplotypes at the nodes T1b1, T1c1 and T1c1a. However, an alternative scenario might be envisioned for haplogroup T1d. Indeed, it encompasses only mtDNAs from Africa. It is noteworthy that our sequences (Figure 1) were the only T1d mtDNAs in our local dataset of 281 T1 samples.

Unfortunately the analysis of GenBank control-region sequences was not of help, since the T1d sub-haplogroup is defined by a mutation in the coding region (np 6235). Thus, it could be hypothesized that this mutation and the derived haplogroup arose shortly after the domestication event in Near Eastern cattle, during its migration process into the African continent, most likely through Egypt. In line with this is the presence of an Egyptian sample in the sub-clade T1d1 (#58) and a coalescence time for T1d (9.4±2.3 ka) that seems slightly lower than that of the other T1 sub-haplogroups.

It should be reminded that our study focuses on mtDNA, which is maternally transmitted. Therefore, a genetic contribution of African wild aurochsen to the formation of modern African cattle breeds [24], [25] cannot be ruled out by our findings, and is compatible with a scenario in which such a genetic input was from male local aurochsen.

It is apparent from our data (Table 1) that the geographical distribution of T1 sub-haplogroups in Ethiopia and Egypt is quite different. Thus, T1c is the most common in Egypt (38.5%) but it is absent in Ethiopia. In contrast T1b encompasses 69.4% of Ethiopian cattle vs 26.9% in Egypt. A possible exaplanation for this finding is that domesticated taurine cattle arrived to Africa from southwest Asia in multiple waves and following independent routes, from the south through Arabia to Somalia and Ethiopia, and from the north through the Isthmuz of Suez to Egypt [26]. Thus, each of these migratory inputs was an oppurtunity for serial founder events to take place, affecting the current sub-haplogorup frequency distribution.

### The “AA" (African-derived American) haplotype is a deep subclade of T1c and is present in Africa

The so called “African-derived American" control-region haplotype (16050–16053–16113–16122–16139–16196–16255) was originally described in Creole cattle, in the Spanish cattle breed Retinta and in one subject of the Lidia breed [16], [17], [21], [22]. Whereas some authors [17] hypothesized an Iberian ancestry for American cattle breed, others [22] argued that Creole cattle might also derive from a direct introduction of African cattle into South America. However, AA haplotypes had never been found in African samples.

In this work, the AA haplotype was analysed by whole mtDNA genome sequencing and turned out to be a sub-clade of T1c, namely T1c1a1 (Figure 1). As expected from previous reports, haplotypes belonging to this sub-clade are observed in samples from South America – Paraguay in our case (#53, 54 and 55) – but the analysis of our local 281 T1 samples highlighted also one animal from Egypt displaying a control region with the diagnostic “AA" mutational motif. Upon complete mtDNA genome sequencing (#52 of Figure 1) the affiliation of this sample to T1c1a1 was confirmed. To our knowledge, this is the first time that the “AA" haplotype is found in an African breed. In summary, T1c1a1 has never been observed in the Near East, it has a rather recent origin (4.9±2.0 ka) and it is present in Africa and Iberia, in addition to the New World.

### Conclusion

The analysis of 64 mitochondrial genomes performed at the level of complete sequence, together with an evaluation of control-region GenBank entries, support the hypothesis that at least 7–8 independent female lineages belonging to haplogroup T1 underwent domestication in the Near East and spread across different areas of the world following human migrations. Our data also suggest that one sub-haplogroup, T1d, might represent a mitochondrial line that has developed in the African continent shortly after the domestication event in the Near East. Finally, we redefined the so-called African-derived American “AA" haplotype as a T1c subclade, namely T1c1a1, and found for the first time an African sequence showing its mutational motif. Thus, our data are compatible with a direct African input in Creole cattle, possibly in addition to the indirect genetic contribution mediated by Iberian cattle.

## Materials and Methods

### Ethics statement

All experimental procedures were reviewed and approved by the Animal Research Ethics Committee of the University of Pavia, Prot. 2/2010 (October 15^th^, 2010), in accordance with the European Union Directive 86/609.

### Samples

A set of 281 T1 mtDNAs was analyzed, encompassing 15 European (12 Italian, 1 French, 1 Portuguese and 1 Turkish), 10 African (3 Egyptian and 7 Ethiopian) and 3 American (Creole cattle from Mexico and Paraguay) breeds. DNAs were purified from peripheral blood according to standard methods.

### Sequence analysis of the mtDNA control region

For all 281 mtDNAs, a PCR fragment of 1138 bp encompassing the control region (nps 15718-517) was sequenced using the oligonucleotide 15757for, 5′ccccaaagctgaagttctat3′, as previously described [8]. Reads covered at least 730 bp, approximately from np 15823 to np 215. Sequences were aligned to the *Bos taurus* Reference Sequence (BRS) [23] using the Sequencher 4.9 software (Gene Codes Corporation).

### Survey of sub-haplogroup diagnostic markers

The presence of sub-haplogroup diagnostic markers was assayed by either sequence or RFLP analysis. The insertion 2055+C (sub-haplogroup T1a) and the transitions at nps 6235 (sub-haplogroup T1d) and 7542 (sub-haplogroup T1b) were assayed by sequence analysis of PCR fragments #3, #5 and #6, respectively, which were produced by following the conditions described in [8]. These PCR fragments were sequenced with oligonucleotides 1915for, 6011for and 7055for, respectively [8].

To evaluate the presence of the G to A transition at np 7542 (sub-haplogroup T1b) a RFLP analysis was also performed for some of the samples. A PCR fragment of 235 bp spanning nps 7335–7569 was amplified with primers Forward 5′tcaaagttaagttacaagtgaaagtcc3′ and Reverse 5′ttcagattgtctctacttcttgtgaat3′as follows: 95°C for 5 minutes followed by 35 cycles at 95°C for 30″, 58°C for 30″, 72°C for 30″ and a final extension step at 72°C for 10 minutes. The Reverse primer carries a mismatch relative to the BRS at the third from last 3′ nucleotide position, which creates a cutting site for *Hinf*I (G/ANTC) when the amplicon carries a G at np 7542 as BRS. The PCR fragment contains another G/ANTC site. Thus, fragments with a G at np 7542 are cut twice by the enzyme and yield three fragments (27 bp, 62 bp, 146 bp). Conversely, when np 7542 is mutated from G to A, one cutting site for *Hinf*I is lost and only fragments of 62 bp and 173 bp are produced. The cutting reaction was performed in a final reaction volume of 20 μl (12.9 μl H_2_O, 2 μl Buffer R 10X, 0.1 μl HinfI 10 U/μl Fermentas, 5 μl PCR product) for 3 hours or O/N at 37°C. Digestion products were separated on a 4% agarose gel.

### Sanger sequencing of mitochondrial genomes

The entire sequence of 26 mtDNAs (Table 2) was determined as previously reported [8]. In brief, a set of 11 overlapping PCR fragments covering the entire mtDNA genome was produced and sequenced by standard dideoxy-sequencing with 32 nested oligonucleotides. To derive individual sequences, raw sequence data were grouped into mtDNA genome contigs and compared to the BRS with the software Sequencher 4.9 (Gene Codes).

### Illumina sequencing of mitochondrial genomes

The remaining 28 mtDNAs were sequenced with an Illumina Genome Analyzer IIx (Table 2). A set of only six overlapping PCR fragments (Table S3) covering the entire mtDNA genome was used in this case. The PCR protocol was as follows: 10 ng of each DNA sample were amplified in 25 μl reaction mixture containing 2.5 mM MgCl_2_, 0.2 mM of each dNTP (Promega), 1X GoTaq Flexi Buffer (Promega), 0.75 U of GoTaq DNA Polymerase (Promega) and 0.3 μM of each primer. The PCR program included an initial step of denaturation at 95°C for 5 minutes followed by 35 cycles of amplification characterized by the following profile: 95°C for 30″, 59°C for 30″, 72°C for 3 minutes and 30″ and a final extension step at 72°C for 10 minutes. For each sample, equimolar quantities of the six PCR products, quantified on agarose gel, were pooled and subsequently purified with the QIAquick PCR purification kit (Qiagen). Around 2.5 μg of each pool were used for the preparation of a sequencing library containing 48 different mtDNA samples (28 belonging to T1 and 20 to other haplogroups).

Briefly, 2.5 μg of each sample were fragmented with 1.7 μl of NEBNext® dsDNA Fragmentase (New England Biolabs) for 2 hours at 37°C in a 50 μl final reaction volume. The reaction was stopped by adding 5 μl EDTA 0.5 M pH 8 and chilling on ice, and DNA purified with the Agencourt® AMPure® XP (Beckman Coulter) system. The subsequent enzymatic reactions of end Repair, 3′ ends adenylation and adapters ligation were performed with NEBNext® DNA Sample Prep Reagent Set 1 (New England Biolabs) following the instructions provided by the manufacturer. After adapters ligation, enrichment PCR was performed using the procedure and reagents provided by the Illumina Paired-End Sample Preparation kit. Each sample was tagged by the addition of a unique Index Sequence at this step. Purified PCR products were pooled into groups of different samples having homogeneous concentrations. Then, the pools were separated on agarose gel and the regions comprised in the range 300–400 bp were excised. Agilent Bioanalyzer was used to quantify each purified pool and to determine the size of its fragments. A final pool was constituted and validated at Agilent Bioanalyzer.

Eight pM of the final pool were loaded on Illumina cBot to perform cluster amplification on a single lane of the flow cell. Finally, sequencing with an Illumina Genome Analyzer IIx (50 bp single reads) was performed.

Tab-delimited text files containing sequence alignment data (SAM files) or their binary version (BAM files) were obtained by aligning raw Illumina sequencing data to the reference sequence (BRS) with the software CLC Genomics Workbench 4. This software was also used to create a report of sequence variants (nucleotide substitutions and indels). The software Tablet [27] was used as an alignment viewer for sequence assemblies in SAM format.

Two mtDNAs (#15 and #31 in Figure 1) previously sequenced with standard dideoxy sequencing were re-sequenced on the Illumina platform as internal controls: in both cases the calling of variants was in total agreement with the outcome of Sanger sequencing (see Table S4).

### T1 phylogeny and time estimates

The tree of Figure 1 is a T1-expanded portion of a complete tree that was rooted using a *Bos grunniens* (yak) and *Bison bison* (American bison) mitochondrial genome, as previously reported [8], [11]. The evolutionary distances were computed using the Maximum Likelihood method (PAML vs. 4.4), together with averaged distance (ρ) of the haplotypes within a clade from the respective root haplotype, accompanied by a heuristic estimate of SE (σ). All positions containing gaps and ambiguous data were eliminated from the dataset. Estimate of the time to the most recent common ancestor for each cluster was calculated using a corrected age estimate of about 3,172 years per substitution in the whole coding region (15,428 bp) [8].

## Supporting Information

Table S1 **List of the 281 control-region haplotypes analyzed in this study.**
(XLS)

Table S2 **List of control-region haplotypes belonging to haplogroup T1 retrieved from GenBank.**
(XLS)

Table S3 **Amplicons and oligonucleotides used for sequencing the whole mitochondrial genome with the Illumina Genome Analyzer IIx.**
(DOC)

Table S4 **Comparison of dideoxy** ***vs*** **Illumina sequencing results for samples CINL14 (#31) and CIN11 (#15).**
(XLS)

Data S1 **The origin of T1 sub-haplogroups and their geographical distribution.**
(DOC)
