# Supplementary material for: Origin and Spread of Bos taurus: New Clues from Mitochondrial Genomes Belonging to Haplogroup T1
Source: PLoS One. 2012 Jun 7;7(6):e38601. doi: 10.1371/journal.pone.0038601 (PMC3369859; doi:10.1371/journal.pone.0038601)
Supplement: Table S3 — Amplicons and oligonucleotides used for sequencing the whole mitochondrial genome with the Illumina Genome Analyzer IIx. (DOC) [file pone.0038601.s003.doc]

| **Fragment #** | **PCR product length (bp)** | **Oligonucleotides 5’-3’^a^** | |
| --- | --- | --- | --- |
| 1 | 2313 | 16272For | TTCTTTCTTCAGGGCCATCT |
|  |  | 2246Rev | TTTCCTTAGATGCACTCCTGTG |
| 2 | 3393 | 1807For | TAGCTGGTTGTCCAGAAAATGA |
|  |  | 5199Rev | TTATGTTGTTTGTGGAGGGAAA |
| 3 | 3504 | 4922For | TATAGCCAATTCCACCACCACT |
|  |  | 8425Rev | GGAGGGTTACAAAGCGATTG |
| 4 | 3371 | 8109For | CATATACTCTCCTTGGTGACATGC |
|  |  | 11479Rev | AATCATAAGGGCGGTTGCTC |
| 5 | 3425 | 11201For | ATCGCAGGCTCCATAGTCCT |
|  |  | 14625Rev | CCAGGAGGGAACCGAAAT |
| 6 | 2646 | 14210For | TTCTGTAGCCATAGCCGTTGTA |
|  |  | 517Rev | AACCTAGAGGGCATTCTCACTG |

**Table S3.** Amplicons and oligonucleotides used for sequencing the whole mitochondrial genome with the Illumina Genome Analyzer IIx.

^a^ The oligonucleotide codes refer to the nucleotide position of the initial (5’) base; For, forward; Rev, reverse.
