# Supplementary material for: Origin and Spread of Bos taurus: New Clues from Mitochondrial Genomes Belonging to Haplogroup T1
Source: PLoS One. 2012 Jun 7;7(6):e38601. doi: 10.1371/journal.pone.0038601 (PMC3369859; doi:10.1371/journal.pone.0038601)
Supplement: Data S1 — The origin of T1 sub-haplogroups and their geographical distribution. (DOC) [file pone.0038601.s005.doc]

**Data S1**

*The T1 sub-haplogroups and their geographical distribution*

The analysis of 64 complete T1 sequences allowed the identification of six sub-haplogroups within haplogroup T1 (Figure 1). The sub-haplogroup T1a in the new phylogenetic tree is defined by the mutation 2055+C. This had already been described [8], associated to 4 sequences. Now this sub-haplogroup is the most represented in the tree and encompasses 28 sequences. Most of them (24) are from Italian breeds, a bias due to the fact that the T1a samples selected for sequencing happened to be mainly Italian. However, when the status of 2055+C within our local dataset of 281 T1 samples was investigated, the majority (65%) of European samples turned out to belong to T1a, whereas 25.3% of the Ethiopians and 26.9% of the Egyptians were classified into such sub-haplogroup, confirming that T1a is more common among European breeds compared with African (Table 1). It should also be noted that for a better understanding of the T1a geographical distribution, the analysis of T1 control regions from GenBank (Table S2) is not useful, as T1a samples are identified by means of the 2055+C insertion, which falls outside the control region.

Sub-haplogroup T1b is composed of 10 new sequences and 2 sequences that were present in the previous tree [8]. They belong to both European and African breeds. We also typed all the 281 T1 subjects of our dataset at the np 7542 and found that 16.3% European samples, 26.9% Egyptians and the majority (69.4%) of Ethiopians group into T1b (Table 1, Table S1). Furthermore, 89.1% of all T1b control regions of the dataset cluster within the subclade T1b1 (diagnostic transition at np 16022). The analysis of GenBank T1 control regions – limited to samples where np 16022 is included in the reads - allows to evaluate the frequency and geographic distribution of putative T1b1 sequences but not those of all T1b sequences since coding region data required to assess the status at np 7542 are missing. Thus, only 334 out of 752 GenBank control regions span np 16022; of those, 131 can be considered as putative T1b1 since they carry the transition at np 16022, although the absence of coding region data cannot exclude the affiliation to other sub-haplogroups. As for their geographical origin, the majority (87) come from Africa (82 from Ethiopia and 5 from Kenya). The others are found in European breeds (Portugal, Spain), American Creole (Colombia, Mexico) and one in a local Chinese breed, Zhaotong.

In the new T1 phylogenetic tree (Figure 1), the sub-branch defined by np 16196 in the previous tree [8] is now comprised within T1c, which is characterized by the control-region transition at np 16122. Like for other sub-haplogroups, the GenBank survey allowed to better assess the geographical distribution of T1c and its sub-clades T1c1, T1c1a and T1c1a1. However, when a control-region sequence displays the transition at np 16122, without 16196 and 16053, its classification within T1c is ambiguous in the absence of coding region data that could classify it within other sub-haplogroups such as T1a, T1b or T1d. As an example, one Ethiopian sample of our local dataset of 281 T1 samples showing 16122 in its CTR belongs to T1b because it also presents the mutation at np 7542. Similarly three Limousin and one Mexican Creole with 16122 share also 2055+C, which means that they belong to sub-haplogroup T1a.

Among the 752 T1 GenBank sequences, 36 turned out to carry the transition at np 16122 without 16053 and 16196, thus likely but not surely belonging to T1c. Their geographical origin is mainly African since 28 subjects are from Egypt, Libya, Tunisia, Kenya, Mozambique, Sudan, Cameroon, Ethiopia, and the other 8 subjects belong to breeds from South America (Mexico, Paraguay), Israele, New Zealand and Italy. Eighty-six GenBank sequences carry both 16122 and 16196, thus belonging to T1c1. Twenty-two of them (from Portugal, Morocco, Tunisia, Libya and Mexico) do not display 16053, while 64 sequences also display the transition at np 16053 diagnostic of T1c1a: 60 of them share the “AA” mutational motif since they also carry 16139 (T1c1a1) and all belong to South American Creole breeds; 4 sequences (two Cuban Creole and two Tunisia Blonde) do not have 16139. Moreover, in the literature the “AA” haplotype was retrieved also in the Spanish cattle breeds Retinta [16,17] and Lidia [21].

The T1d sub-clade and has been defined for the first time in the new T1 phylogenetic tree described here (Figure 1). It looks like an only-African sub-clade since no T1d samples were retrieved in Europe (Table 1), however it should be noted that a detailed analysis of its geographic distribution within the sequences available in GenBank is hindered by the fact that the T1d definition is based on the presence of a mutation in the coding region (np 6235) which is typically missing in GenBank control-region entries. This means that T1d could be found also elsewhere, possibly even in non African subjects, even if at very low frequencies.

Among the GenBank control regions, 19 share the mutational motif 16113-16255 with a reversion at 16050 and without other control-region diagnostic mutations such as 16022, 16122 and 16196. This is the hallmark of the sub-haplogroup T1e. Their geographical origin is European (Italy, Portugal, Spain), African (Nigeria, Sudan, Mozambique, Ethiopia) and two were retrieved in Bonsmara (South African breed imported to Argentina) and Cuban Creole subjects.

Finally, 28 GenBank control regions are distinguished by the mutational motif 16050-16255 with a reversion at np 16113, diagnostic of the newly defined sub-haplogroup T1f. Interestingly, this mutational motif is predominant among T1 control regions from local breeds of China, Japan and Korea, and is also retrieved in Africa and with a very low frequency also in Europe.
